# RapidArc dynamic versus RapidArc: A multi anatomical‐site dosimetric evaluation

**DOI:** 10.1002/acm2.70404

**Published:** 2026-01-14

**Authors:** Aram Rostami, Carole Naim, Renilmon Pottanplackal Sukumaran, Mohammad Usman, Abbass Yousef Mkanna, Alla Fuad Al‐Sabahi, Ahamed Basith, Shelton Chuck, Mojtaba Barzegar, Bevan Orville Peltier, Bassim Aklan, Samaneh Baradaran, Satheesh Prasad Paloor, Rabih Wafiq Hammoud

**Affiliations:** ^1^ Radiation Oncology Department National Center for Cancer Care and Research Doha Qatar; ^2^ Nuclear Science & Technology Research Institute Tehran Iran

**Keywords:** RapidArc, RapidArc dynamic, volumetric modulated arc therapy

## Abstract

**Background:**

RapidArc (RA) has advanced VMAT delivery; however, challenges remain in achieving optimal organ‐at‐risk (OAR) sparing in complex cases. RapidArc Dynamic (RAD), a next‐generation Volumetric Modulation Arc Therapy (VMAT) technique, incorporates dynamic collimator rotation, embedded static fields, and a faster optimizer to enhance dose modulation. This study compares dosimetric outcomes between conventional RA and RAD across four anatomical sites.

**Methods:**

A retrospective analysis was conducted on 40 patients (10 each for breast, lung SBRT, prostate, and head and neck cancers). Each case was initially treated with RA and subsequently replanned using RAD in Eclipse v18.1. Plans were evaluated using dose–volume histograms (DVH), and paired t‐tests assessed differences in target coverage and OAR sparing (*p* < 0.05).

**Results:**

RAD maintained equivalent target coverage compared to RA across all sites. Significant OAR sparing was observed with RAD, including reduced mean heart and contralateral lung doses in breast cases, lower rectal and bladder doses in prostate cases, improved conformity and reduced spinal cord and lung doses in lung SBRT, and superior sparing of parotids, cochleae, and mucosal structures in head and neck cases.

**Conclusion:**

RAD offers superior OAR sparing and improved dose conformity without compromising target coverage compared to conventional RA. These results support RAD's clinical adoption, particularly for anatomically complex or high‐precision treatments. Further prospective studies are needed to assess the long‐term clinical benefits.

## 1 INTRODUCTION

Volumetric modulated arc therapy (VMAT) has transformed the field of radiation oncology by enabling precise and efficient delivery of radiation doses. Unlike traditional static‐beam intensity‐modulated radiotherapy (IMRT), VMAT delivers radiation continuously as the machine's gantry rotates around the patient, allowing for highly conformal treatment in a shorter time.^1^ One of the earliest and most widely used VMAT techniques is RapidArc (developed by Varian Medical Systems, Palo Alto, CA), which coordinates changes in multileaf collimator (MLC) positions, gantry speed, and dose rate to shape the dose dynamically during treatment.^2^ However, in complex clinical situations—such as head and neck tumors or breast cancers with nodal involvement, conventional RapidArc (RA) may struggle to maintain sharp dose gradients or adequately limit low‐dose exposure to surrounding healthy tissues.^2^, ^3^ To address these limitations, RapidArc Dynamic (RAD) was introduced as part of newer software (Eclipse v18.1) and LINAC control system upgrades (TrueBeam v4.1+). RAD represents a significant evolution of the original RapidArc technique. Unlike conventional RA, which relies solely on dynamic arcs, RAD integrates multiple advanced delivery strategies within a single plan, including: directional control of IMRT and efficiency of VMAT delivery in a single treatment field, ability to strategically pause the gantry during an arc rotation for enhanced modulation at optimal beam angles and dynamically adjust collimator rotation to improve dose conformity and organ‐at‐risk sparing during delivery.^4^

In addition to these mechanical enhancements, RAD also features a new, faster optimization algorithm, which reduces planning time while maintaining dosimetric quality. RAD introduces another practical improvement—auto skin flash tools that help ensure adequate coverage of superficial targets, particularly in breast cases. Moreover, the new Photon Optimizer (PO) used in RAD planning offers an additional level of flexibility: within the optimization window, the user can control the relative weighting between static fields and arc components. This includes options such as balanced mode, arc‐dominant, or static field‐dominant settings, allowing further customization of plan modulation based on clinical objectives.^4^

This hybrid planning and delivery approach using dynamic collimator during arc offers enhanced flexibility in dose modulation and is particularly beneficial for treating anatomically complex or irregular targets—such as concave planning target volumes (PTVs) or tumors located near critical serial organs‐at‐risk (OARs), including the optic nerves and spinal cord.^4^ Preliminary hypotheses and vendor‐reported data suggest that RAD may provide improved OAR sparing, sharper dose fall‐off, and enhanced PTV coverage and conformity. However, multi‐anatomical‐site dosimetric comparisons remain limited.^4^

We hypothesized that RAD's added capabilities—particularly dynamic collimator rotation, incorporation of static fields during arc delivery, and built‐in skin flash for breast planning—could result in superior PTV coverage and OAR sparing compared to conventional RA, especially in anatomically complex cases. To evaluate this hypothesis, we conducted a retrospective dosimetric study in which newly generated RAD plans were compared against the clinically delivered RA plans across four anatomical treatment sites (Breast, Lung SBRT, Prostate, and Head & Neck). A systematic dose–volume histogram (DVH)‐based analysis was performed, and paired t‐tests were used to determine the statistical significance of dosimetric differences between the two planning approaches.

## 2 MATERIALS AND METHODS

### 2.1 Study design and patient selection

Following institutional review board approval, this retrospective multi‐anatomical‐site dosimetric study was conducted to compare radiation treatment plans generated using RA and RAD techniques. A total of 40 patients were included across four anatomical sites: Breast cancer (with internal mammary node involvement): 10 patients (5 left‐sided, 5 right‐sided), Lung SBRT: 10 patients, Prostate cancer (with nodal involvement): 10 patients and Head and Neck cancer (with nodal involvement): 10 patients.

For HN cases, treatment plans incorporated simultaneous integrated boost (SIB) over 33 fractions, covering three target volumes: high‐risk (70 Gy), medium‐risk (60 Gy), and low‐risk (54 Gy). For OARs, standard institutional dose thresholds were applied uniformly rather than linking constraints to specific SIB dose levels (70/60/54 Gy). Identical constraints and weightings were applied for both RA and RAD optimizations. These constraints were pre‐specified during optimization and applied consistently across all cases. For prostate cases, plans were delivered over 28 fractions, incorporating two or three target levels: a high‐risk volume (70 Gy) covering the prostate and seminal vesicles, a nodal volume (50.4 Gy), and, when indicated, PSMA PET‐positive bone metastases (64.4 Gy). For breast cases, treatments were planned over 15 fractions, including coverage of the supraclavicular and internal mammary node (IMN) PTVs as part of the target volume. This retrospective analysis was conducted using fully anonymized treatment planning data, with no impact on patient care.

Each patient case was initially planned using the RA technique and subsequently replanned using the RAD technique, enabling direct intra‐patient dosimetric comparisons.

### 2.2 Technology and software

RAD treatment planning was performed using the Eclipse Treatment Planning System (TPS) v18.1 (Varian Medical Systems, Palo Alto, CA), which features RAD capabilities, including dynamic collimator rotation and embedded static beam segments. For all RAD plans, the STAMP weighting parameter was set to −1 (‘Arc’ weighting) on the optimization window of RAD (−2: Arc Dominant, −1: Arc, 0: Balanced, +1: Static, +2: Static Dominant), indicating an arc‐leaning modulation preference during optimization. All static field angles were user‐specific and selected by the planner, based on the principle of choosing gantry angles that provided maximum geometrical view of the target and minimum geometrical view of adjacent critical OARs. All RA plans, originally generated in Eclipse v16.1, were re‐optimized and recalculated in Eclipse v18.1 to ensure consistency with RAD planning for comparative analysis. All dose calculations for RA and RAD were performed using the Acuros XB (AXB) version 18.1 algorithm with heterogeneity correction enabled, employing a calculation grid size of 2.5 mm for all sites, except for Lung SBRT cases, where a finer 1.25 mm grid size was used to improve dose calculation accuracy in small, high‐gradient regions. For all Lung SBRT cases, motion management was performed using 4DCT simulation to account for respiratory motion, and internal target volumes (ITVs) were delineated accordingly. The same CT datasets were used for both RA and RAD planning across all anatomical sites to ensure consistency and minimize setup‐related differences. Treatment delivery was simulated on Varian TrueBeam LINACs (v4.1) equipped with millennium multileaf collimators.

### 2.3 Planning techniques and geometry

RA plans utilized conventional VMAT delivery with static collimator angles and no static beam segments during arc delivery.

For both RA and RAD plans, maximum MLC leaf speed was restricted to 2.5 cm/s, and jaw tracking was enabled with a maximum jaw speed of 2.5 cm/s. The maximum field width was limited to 15 cm in the X‐direction to respect MLC leaf span constraints. For RAD plans, dynamic collimator rotation was permitted with a maximum rotation speed of 9°/s.

RAD plans incorporated advanced features, including: dynamic collimator rotation during beam‐on, embedded static fields within arcs, enhanced PO algorithm for faster and more effective optimization and automatic skin flash generation for breast cases. Plan geometry details by site are summarized in Table 1.

**TABLE 1:** Plan geometry comparison between RapidArc dynamic (RAD) and RapidArc (RA).

Parameter	Breast (RAD)	Breast (RA)	Head & Neck (RAD)	Head & Neck (RA)	Prostate (RAD)	Prostate (RA)
Arc span	305–175 (Left Breast) 55–185 (Right Breast)	2 Arcs (305–175 and reverse) 2 Arcs (55–185 and reverse)	1 Full Arc	2 Full Arcs	2 Full Arcs	2 Full Arcs
Static fields	305, 330, 122, 175 (Left) 55, 30, 206, 237 (Right)	N/A	4 Static Fields per arc	N/A	2 Static Fields (Arc 1) 2 Static Fields (Arc 2)	N/A
Collimator	Optimized	Static	Optimized	Static	Optimized	Static
Static and arc weighting	Balanced	N/A	(−1) Arc	N/A	(−1) Arc	N/A
Total monitor units (MU)	912	731	820	710	1042	910
Estimated planning time	160 min	205 min	150 min	155 min	135 min	142 min
Dose calculation algorithm	Acuros XB (V.18.1)	Acuros XB (V.18.1)	Acuros XB (V.18.1)	Acuros XB (V.18.1)	Acuros XB (V.18.1)	Acuros XB (V.18.1)
Optimization algorithm	Photon Optimizer (V18.1)	Photon Optimizer (V18.1)	Photon Optimizer (V18.1)	Photon Optimizer (V18.1)	Photon Optimizer (V18.1)	Photon Optimizer (V18.1)

**Note1**: Lung SBRT cases employed individualized arc spans and static field angles based on tumor location and proximity to critical structures; plan complexity was adjusted case by case. For both RA and RAD, the number of arcs ranged between 3 and 6 (full or partial arcs), while RAD plans incorporated an additional 1–2 static fields per arc. All treatment plans were coplanar, with the couch fixed at 0°, and collimator angles were left to be optimized by the TPS for RAD planning. **Note 2**: The arc/static weighting scheme (e.g., balanced, arc‐dominant) for RAD plans was determined through iterative optimization by user. Different configurations were tested, and the final weighting was selected based on achieving the best compromise between target coverage and OAR sparing for each case. **Note 3**:’ (−1) Arc ’ refers to the RAD STAMP weighting level (Arc setting) on the optimization scale.

### 2.4 Plan evaluation metrics

For both RA and RAD plans, identical optimization objectives and OAR dose constraints were applied, with the same relative weighting factors in the Photon Optimizer. This ensured that observed differences reflected the planning technique rather than differences in optimization priorities

Each plan was done and reviewed by a multidisciplinary team consisting of two senior medical physicists per anatomical site. Evaluation was based on dose volume histogram comparison.

Following plan generation for both RA and RAD, we conducted a paired *t*‐test (significance: *p* < 0.05) to compare the target Coverage (the percentage of the target volume receiving 95% of the prescribed dose V95%), OAR Doses (the dose delivered to each OAR, aiming to identify potential reductions in dose exposure) and conformity index (CI) using Paddick formula ^5^ for SBRT cases. Primary endpoints per site were pre‐specified based on clinical relevance. P‐values for these were adjusted using Holm–Bonferroni; all other comparisons were considered exploratory.

## 3 RESULTS

### 3.1 Breast

A comparative analysis of RAD and RA plans for breast cases demonstrated that both techniques achieved comparable dose coverage of the target volumes, with no statistically significant differences in optimized planning target Volume (OPTV) V95 or V105 and supraclavicular planning target volume (PTV‐SC) coverage (see Table 2).

**TABLE 2:** Dosimetric comparison between RapidArc dynamic (RAD) and conventional RapidArc (RA) for breast cancer cases involving IMN and SCF regions. Optimized Planning Target Volume (OPTV): Breast PTV cropped 5 mm from the skin; PTV‐SC: Supraclavicular planning target volume.

OARs	Dose constraint	Left breast with IMN and SCF			Right breast with IMN and SCF			Total		
		RAD (Average)	RA (Average)	Difference (%)	RAD (Average)	RA (Average)	Difference (%)	RAD (Average)	RA (Average)	Difference (%)
Heart	V23 Gy (%)	0.14	0.17	−16.48	0.14	0.18	−12.50	0.139	0.173	−19.47
Heart	D_mean_ (Gy)	2.668	3.518	−24.17	1.940	2.681	−26.51	2.319	3.099	−25.18
Breast contralateral	D_mean_ (Gy)	2.048	2.752	−25.58	2.176	2.721	−20.02	2.112	2.736	−22.82
Lung ipsilateral	D_mean_ (Gy)	10.056	10.597	−5.11	9.328	10.09	−7.61	9.692	10.347	−6.33
Lung ipsilateral	V18 Gy (%)	21.12	21.32	−1.0	18.82	20.10	−6.37	19.97	20.71	−3.57
Lung ipsilateral	V5 Gy (%)	50.36	57.96	−13.11	48.8	52.24	−8.5	49.08	55.10	−10.93
Lung contralateral	D_mean_ (Gy)	2.088	2.587	−19.29	1.652	1.912	−13.58	1.870	2.249	−16.86
Spinal cord	D_max_ (Gy)	10.80	15.39	−29.85	11.322	16.40	−30.98	11.062	15.901	−30.43
Humeral head	D_max_ (Gy)	19.44	21.10	−7.76	20.769	24.334	−18.02	20.107	23.218	−13.4
OPTV	V95 (%)	96.82	96.58	0.25	98.56	97.94	0.63	97.69	97.26	0.44
OPTV	V105 (%)	10.08	7.76	29.90	10.36	10.28	0.78	10.22	9.02	13.3
PTV‐SC	V95 (%)	98.54	98.12	0.43	98.22	98.22	0.0	98.38	98.17	0.21
PTV‐SC	V105 (%)	1.73	2.06	−16.52	2.24	1.99	15.2	1.483	2.03	−26.9

When considering pre‐specified primary endpoints, RAD yielded a significant reduction in the maximum spinal‐cord dose after Bonferroni correction (p = 0.003 < 0.0167). The mean heart dose was also reduced by approximately 25%, with a corrected *p* = 0.024, representing a strong trend toward significance, while the ipsilateral lung V20 did not differ significantly between techniques (*p* = 0.48), see Table 3.

**TABLE 3:** Primary dosimetric endpoints for breast cases—bonferroni‐corrected comparison between RapidArc dynamic (RAD) and conventional RapidArc (RA).

Endpoint	RAD (Average)	RA (Average)	Δ (%)	Raw *p*‐value	Bonferroni Corrected *p*‐value (×3)	Significant (*α* = 0.05)	Significant after correction (*α* = 0.05/3 = 0.0167)
Heart D_mean_ (Gy)	2.319	3.099	−25.18	0.008	0.024	Yes	No
Ipsilateral lung V20 Gy (%)	19.970	20.710	−3.57	0.480	1.000	No	No
Spinal cord D_max_ (Gy)	11.062	15.901	−30.43	0.001	0.003	Yes	Yes

Across exploratory endpoints, RAD consistently demonstrated favorable dosimetric trends. Notably, mean doses to the contralateral breast and contralateral lung were reduced by ∼23% and ∼17%, respectively (*p* = 0.009 for both), and the ipsilateral lung V5 Gy decreased by ∼11% (*p* = 0.008). These reductions reflect lower low‐dose exposure in RAD plans, particularly in the thoracic region adjacent to the heart and lungs. No statistically meaningful differences were observed for the humeral head, ipsilateral lung V18 Gy, or target‐volume hotspot parameters (OPTV V105%), see Table 4.

**TABLE 4:** Exploratory dosimetric endpoints for breast cases—Bonferroni‐corrected comparison between RapidArc dynamic (RAD) and conventional RapidArc (RA).

Endpoint (Exploratory)	RAD (Average)	RA (Average)	Δ (%)	Raw p‐value	Interpretation
Heart V23 Gy (%)	0.139	0.173	−19.47	0.390	No clear difference
Contralateral Breast D_mean_ (Gy)	2.112	2.736	−22.82	0.009	Directionally favors RAD
Ipsilateral Lung D_mean_ (Gy)	9.692	10.347	−6.33	0.098	No clear difference
Ipsilateral Lung V5 Gy (%)	49.080	55.100	−10.93	0.008	Directionally favors RAD
Contralateral Lung D_mean_ (Gy)	1.870	2.249	−16.86	0.009	Directionally favors RAD
Humeral Head D_max_ (Gy)	20.107	23.218	−13.40	0.230	No clear difference
OPTV V95 (%)	97.690	97.260	0.44	0.270	No clear difference
OPTV V105 (%)	10.220	9.020	13.30	0.630	No clear difference
PTV‐SC V95 (%)	98.380	98.170	0.21	0.250	No clear difference
PTV‐SC V105 (%)	1.483	2.030	−26.90	0.980	No clear difference

**FIGURE 1:**
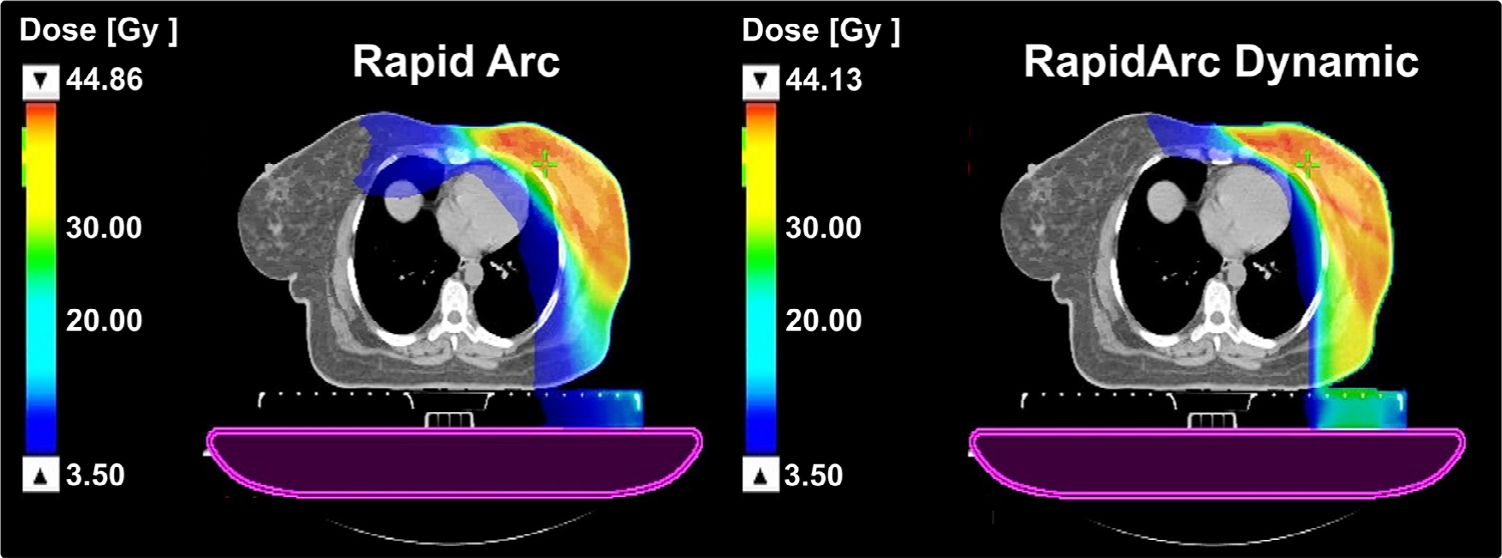
Comparison of the 3.5 Gy isodose distribution between RapidArc and RapidArc dynamic plans for a left breast case.

Collectively, these results confirm that while target coverage remains equivalent, RAD provides clinically relevant improvements in OAR sparing—especially in lowering spinal‐cord and cardiac exposures—with consistent directional benefits for other thoracic structures. Figure 1 illustrates a representative comparison of the 3.5 Gy isodose distribution between RA and RAD plans for a left‐sided breast case, highlighting improved conformity and reduced low‐dose spillage with RAD.

### 3.2 Prostate

The comparison between RAD and RA plans for prostate cancer demonstrated that both techniques achieved comparable target coverage across all PTVs, with no statistically significant differences in V95% for PTV5040, PTV6000, PTV6500, and PTV7000 (*p* > 0.05; Table 5).

**TABLE 5:** Dosimetric comparison between RapidArc dynamic (RAD) and conventional RapidArc (RA) for prostate cancer cases—primary and exploratory endpoints with Bonferroni‐corrected analysis.

Endpoint	Category	RAD (Average)	RA (Average)	Δ (%)	Raw p‐value	Bonferroni Corrected p‐value (×3)	Significant (α = 0.05)	Significant After Correction (α = 0.05/3 = 0.0167)	Interpretation
Rectum V45 Gy (%)	Primary	21.6	26.08	−17.0	0.005	0.015	Yes	Yes	Significant reduction with RAD
Rectum V55 Gy (%)	Primary	12.7	14.19	−10.4	0.008	0.024	Yes	Trend (> 0.0167)	Reduction trend
Bladder V55 Gy (%)	Primary	12.8	14.65	−12.6	0.008	0.024	Yes	Trend (> 0.0167)	Reduction trend
Rectum V60 Gy (%)	Exploratory	8.8	10.19	−13.7	0.070	–	No	–	No difference
Rectum D10 cc (Gy)	Exploratory	50.89	54.18	−6.1	0.040	–	Yes	–	Directionally favors RAD
Rectum D_max_ (Gy)	Exploratory	68.09	68.62	−0.8	0.180	–	No	–	No difference
Bladder V65 Gy (%)	Exploratory	7.4	8.11	−8.4	0.090	–	No	–	No difference
Bladder D_max_ (Gy)	Exploratory	70.50	69.62	+1.3	0.140	–	No	–	No difference
Femoral Head L D_mean_ (Gy)	Exploratory	9.46	15.95	−40.7	0.002	–	Yes	–	Favors RAD
Femoral Head L D_max_ (Gy)	Exploratory	39.46	42.29	−6.7	0.025	–	Yes	–	Favors RAD
Femoral Head R D_mean_ (Gy)	Exploratory	9.16	15.11	−41.2	0.001	–	Yes	–	Favors RAD
Femoral Head R D_max_ (Gy)	Exploratory	35.49	39.54	−10.2	0.043	–	Yes	–	Favors RAD
Bone Marrow V10 Gy (%)	Exploratory	54.2	60.0	−10.3	0.006	–	Yes	–	Favors RAD
Bone Marrow V20 Gy (%)	Exploratory	41.9	45.41	−7.8	0.009	–	Yes	–	Favors RAD
Bone Marrow V40 Gy (%)	Exploratory	12.1	12.2	−0.5	0.9	–	No	–	No difference
Peritoneum V45 Gy (cc)	Exploratory	83.8	91.32	−8.2	0.008	–	Yes	–	Favors RAD
PTV 5040 V95 (%)	Exploratory	97.9	98.81	−1.0	0.120	–	No	–	Equivalent coverage
PTV 6000 V95 (%)	Exploratory	98.9	98.16	+0.8	0.120	–	No	–	Equivalent coverage
PTV 6500 V95 (%)	Exploratory	99.2	97.12	+2.2	0.180	–	No	–	Equivalent coverage
PTV 7000 V95 (%)	Exploratory	97.9	98.22	−0.4	0.290	–	No	–	Equivalent coverage

For the pre‐specified primary endpoints, RAD plans achieved a statistically significant reduction in rectal V45 Gy (*p* = 0.005; Bonferroni‐corrected *p* = 0.015 < 0.0167). Rectum V55 Gy and Bladder V55 Gy also decreased by ≈10%–13%, showing trends toward significance after correction (corrected *p* = 0.024), see Table 5.

Across exploratory endpoints, RAD showed consistent dosimetric advantages. Rectum D10 cc decreased by ≈6% (*p* = 0.040), and although V60 Gy reduction did not reach statistical significance (*p* = 0.07), the direction favored RAD. Significant sparing was observed for both left and right femoral heads, with >40% reductions in mean dose and ≈7%–10% reductions in maximum dose (*p* < 0.05). Bone‐marrow sparing was also superior, with statistically significant decreases in V10 Gy (*p* = 0.006) and V20 Gy (*p* = 0.009), while V40 Gy remained comparable between techniques. The peritoneal V45 Gy was reduced by ≈8% (*p* = 0.008), further supporting improved normal‐tissue sparing with RAD, see Table 5.

**FIGURE 2:**
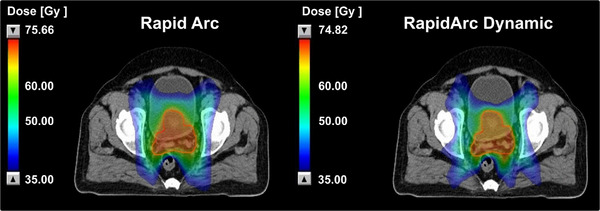
Comparison of the 35 Gy (50% of prescription) isodose distribution between RapidArc and RapidArc dynamic plans for a prostate case.

Overall, RAD maintained equivalent PTV coverage while achieving clinically meaningful reductions in rectal, bladder, femoral‐head, bone‐marrow, and peritoneal doses. These findings indicate lower normal‐tissue doses with RAD for the evaluated structures without compromising target conformity or dose homogeneity. Figure 2 illustrates a representative 35 Gy (50% of prescription) isodose comparison between RA and RAD plans for a typical prostate case.

### 3.3 Lung SBRT

The comparison between RapidArc Dynamic (RAD) and conventional RapidArc (RA) plans for lung SBRT demonstrated that both techniques achieved comparable target coverage, with no statistically significant differences in PTV V95% (*p* = 0.81) or V98% (*p* = 0.075; Table 6). Although V98% was slightly higher in RAD plans, this difference did not reach statistical significance.

**TABLE 6:** Dosimetric comparison between RapidArc dynamic (RAD) and conventional RapidArc (RA) for lung SBRT cases—primary and exploratory endpoints with Bonferroni‐corrected analysis (PTV: Planning Target Volume; V95 (%): Percentage of the target volume receiving at least 95% of the prescribed dose; V5 Gy (%): Percentage of the organ receiving at least 5 Gy).

Endpoint	Category	RAD (Average)	RA (Average)	Δ (%)	Raw p‐value	Bonferroni corrected p‐value(×2)	Significant (α = 0.05)	Significant After Correction (α = 0.05/2 = 0.025)	Interpretation
Conformity Index (CI)	Primary	0.84	0.77	8.3	0.008	0.016	Yes	Yes	Improved conformity with RAD
Spinal Cord D_max_ (Gy)	Primary	9.470	13.493	−29.8	0.004	0.008	Yes	Yes	Significant reduction with RAD
PTV V95 (%)	Exploratory	94.2	93.8	3.1	0.81	–	No	–	Equivalent coverage
PTV V98 (%)	Exploratory	85.8	82.5	3.8	0.075	–	No	–	Comparable
Heart D_mean_ (Gy)	Exploratory	1.150	2.10	−45.2	0.001	–	Yes	–	Favors RAD
Lung D_mean_ (Gy)	Exploratory	4.130	5.680	−27.3	0.040	–	Yes	–	Favors RAD
Lung V5 Gy (%)	Exploratory	22.8	27.1	−15.1	0.035	–	Yes	–	Favors RAD
Lung V10 Gy (%)	Exploratory	15.9	19.3	−17.6	0.033	–	Yes	–	Favors RAD
Lung V20 Gy (%)	Exploratory	7.8	9.2	−15.2	0.034	–	Yes	–	Favors RAD

For the primary endpoints, RAD plans achieved a significantly improved Conformity Index (CI) according to the Paddick formula^5^ (*p* = 0.008; Bonferroni‐corrected *p* = 0.016 < 0.025), confirming superior dose conformity around the target. Similarly, the maximum dose to the spinal cord was significantly reduced with RAD (*p* = 0.004; Bonferroni‐corrected *p* = 0.008), indicating effective spinal‐cord sparing after multiple‐comparison adjustment, see Table 6.

Across exploratory endpoints, RAD also showed consistent normal‐tissue sparing. The mean heart dose was markedly lower in RAD plans (1.15 Gy vs. 2.10 Gy; *p* = 0.001), and the mean lung dose was reduced by approximately 27% (*p* = 0.040). Significant decreases were also observed in Lung V5 Gy, V10 Gy, and V20 Gy (*p* = 0.035, 0.033, and 0.034, respectively), demonstrating reduced low‐ and intermediate‐dose spillage to healthy lung tissue, see Table 6.

**FIGURE 3:**
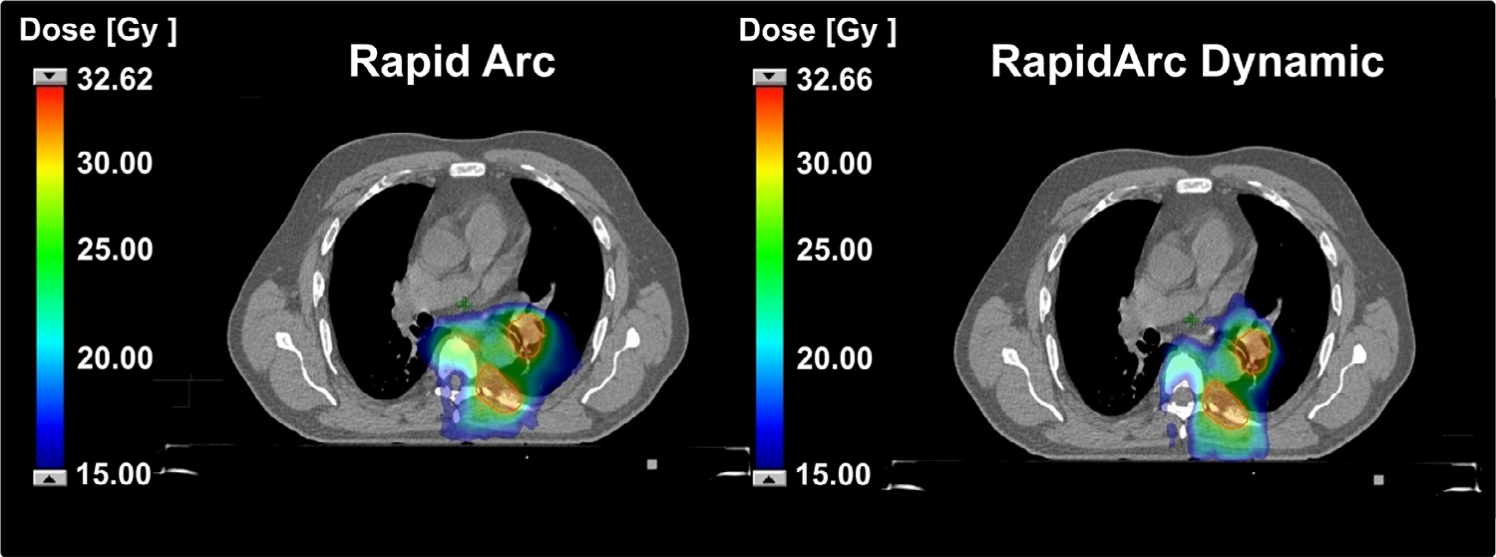
Comparison of 50% of prescription isodose distribution between conventional RapidArc and RapidArc dynamic plans for a lung SBRT.

Overall, RAD planning provided enhanced conformity and superior OAR sparing while maintaining equivalent PTV coverage in lung SBRT. For the ten analyzed cases, treatments were prescribed per institutional protocols, ranging from 24 Gy in three fractions to 40 Gy in 5 fractions, depending on tumor size and proximity to critical structures. Figure 3 illustrates a representative lung SBRT case, where the RAD plan showed improved conformity and reduced heart and lung dose spillage compared with the conventional RA plan.

### 3.4 Head and neck

The comparison between RapidArc dynamic (RAD) and conventional RapidArc (RA) plans for head‐and‐neck cases demonstrated similar target coverage across all planning target volumes (PTVs), with no statistically significant differences observed (*p* > 0.05; Table 7).

**TABLE 7:** Dosimetric comparison between RapidArc Dynamic (RAD) and conventional RapidArc (RA) for head‐and‐neck cases—primary and exploratory endpoints with Bonferroni‐corrected analysis (PTV: Planning target volume; Dmean: Mean dose; Dmax: Maximum dose; PRV: Planning organ‐at‐risk volume).

Endpoint	Category	RAD (Average)	RA (Average)	Δ (%)	Raw p‐value	Bonferroni Corrected p‐value (×3)	Significant (α = 0.05)	Significant After Correction (α = 0.05/3 = 0.0167)	Interpretation
Parotid Left D_mean_ (Gy)	Primary	24.3	26.1	−9.7	0.010	0.030	Yes	Trend (> 0.0167)	Reduction trend
Parotid Right D_mean_ (Gy)	Primary	23.6	26.9	−14.2	0.010	0.030	Yes	Trend (> 0.0167)	Reduction trend
Spinal Cord PRV D_max_ (Gy)	Primary	40.5	42.0	−8.6	0.020	0.060	Yes	No	Slight reduction
Mucosal Constrictors D_mean_ (Gy)	Exploratory	39.5	42.7	−9.2	0.010	–	Yes	–	Favors RAD
Lips D_mean_ (Gy)	Exploratory	20.3	26.1	−23.4	0.010	–	Yes	–	Significant reduction
Larynx D_mean_ (Gy)	Exploratory	42.6	45.9	−8.2	0.020	–	Yes	–	Favors RAD
Esophagus D_mean_ (Gy)	Exploratory	22.5	27.1	−18.2	0.010	–	Yes	–	Favors RAD
Oral Cavity D_mean_ (Gy)	Exploratory	38.6	47.2	−15.1	0.010	–	Yes	–	Favors RAD
Brainstem PRV D_max_ (Gy)	Exploratory	57.5	60.0	−4.6	0.020	–	Yes	–	Reduced D_max_
Optic Nerves PRV D_max_ (Gy)	Exploratory	51.0	51.3	−0.6	0.800	–	No	–	No difference
Optic Chiasm PRV D_max_ (Gy)	Exploratory	52.5	54.5	−5.6	0.040	–	Yes	–	Trend toward reduction
Cochlea Left D_mean_ (Gy)	Exploratory	33.6	39.4	−14.9	0.010	–	Yes	–	Favors RAD
Cochlea Right D_mean_ (Gy)	Exploratory	34.5	40.4	−14.8	0.010	–	Yes	–	Favors RAD
Lens Left D_max_ (Gy)	Exploratory	7.6	9.4	−20.0	0.010	–	Yes	–	Significant reduction
Lens Right D_max_ (Gy)	Exploratory	7.5	9.1	−18.5	0.010	–	Yes	–	Significant reduction
Eye Left D_max_ (Gy)	Exploratory	40.5	42.5	−4.7	>0.05	–	No	–	No difference
Eye Right D_max_ (Gy)	Exploratory	39.5	41.4	−4.6	>0.05	–	No	–	No difference

For the primary endpoints, the mean dose to the left and right parotid glands was reduced by 9.7% and 14.2%, respectively, in RAD compared to RA plans (*p* = 0.010 for both). After Bonferroni correction (*m* = 3; adjusted *α* = 0.0167), both parameters remained trending toward significance, indicating a consistent improvement in parotid sparing. The maximum dose to the spinal cord PRV decreased by 8.6% (*p* = 0.020), demonstrating a meaningful though non‐significant reduction after correction, see Table 7.

Across exploratory endpoints, RAD plans consistently provided improved sparing of critical structures. The mean dose to the mucosal constrictors was reduced by 9.2% (*p* = 0.010), and the mean lip dose decreased by 23.4% (*p* = 0.010). Further reductions were observed for the larynx (−8.2%, *p* = 0.020), esophagus (−18.2%, *p* = 0.010), and oral cavity (−15.1%, *p* = 0.010). The maximum doses to the brainstem and spinal cord PRVs were lower by 4.6% and 8.6%, respectively (both *p* ≈ 0.02).

No significant differences were detected for the optic nerves (*p* > 0.05), while the optic chiasm PRV showed a trend toward reduction (−5.6%, *p* = 0.040). The mean cochlear doses were substantially reduced by 14.9% on the left and 14.8% on the right (*p* = 0.010), and the lens maximum doses decreased by 20.0% and 18.5% on the left and right sides, respectively (*p* = 0.010), see Table 7.

**FIGURE 4:**
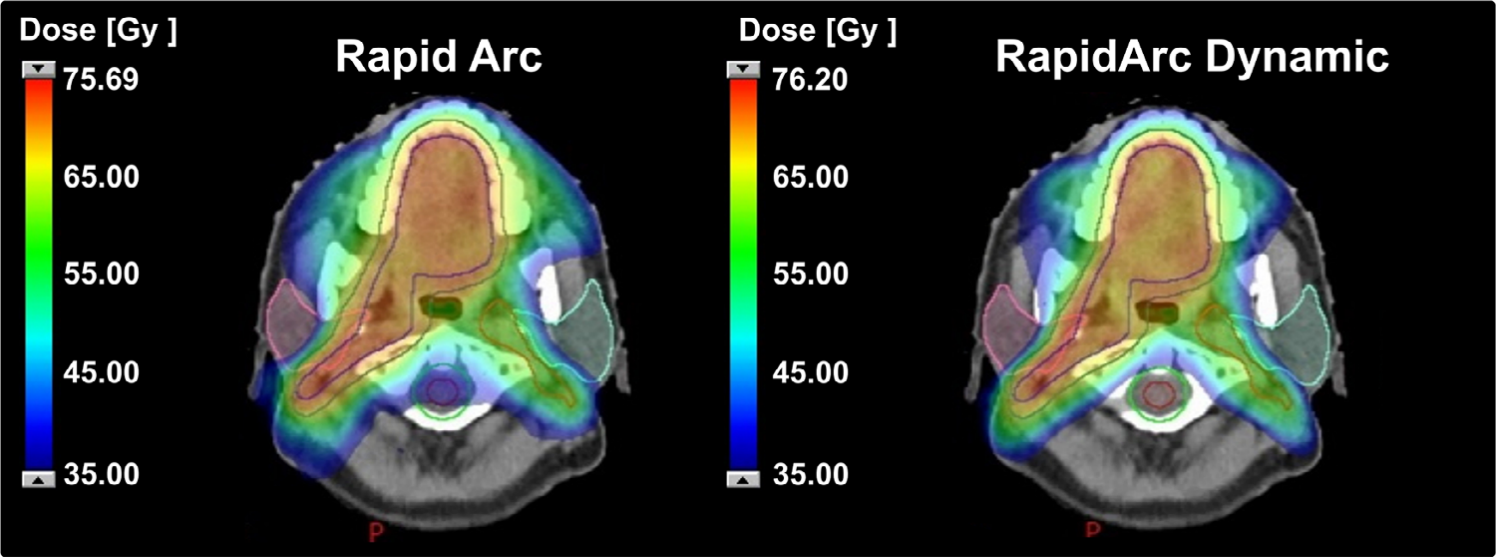
Comparison of the 35 Gy (50% of prescription) isodose distribution between conventional RapidArc and RapidArc dynamic plans for a head and neck case.

Overall, RAD planning achieved improved sparing of multiple organs‐at‐risk while maintaining equivalent PTV coverage in head‐and‐neck cases. The most notable reductions were observed for the parotid glands, lips, cochleae, esophagus, oral cavity, larynx and lenses; although parotids and spinal cord dose reductions were clinically meaningful, they did not reach statistical significance after Bonferroni correction and should be interpreted as trends. Figure 4 illustrates the 35 Gy (50% prescription) isodose distribution comparison between RA and RAD plans for a representative head‐and‐neck case, demonstrating reduced low‐dose spillage and improved OAR sparing with the RAD technique.

## 4 DISCUSSION

This dosimetric study demonstrates that RapidArc Dynamic (RAD) achieves superior treatment plan quality compared to conventional RapidArc (RA) across breast, prostate, lung SBRT, and head and neck cancer cases. RAD consistently enhanced organ‐at‐risk (OAR) sparing and dose conformity while maintaining equivalent target coverage, underscoring its potential to advance precision in volumetric modulated arc therapy (VMAT).

For breast cancer, RAD significantly reduced mean doses to the heart, contralateral breast, and contralateral lung, alongside lower ipsilateral lung V5 Gy exposure. These improvements are clinically critical, as minimizing low‐dose spillage to healthy tissues is associated with reduced risks of cardiopulmonary complications and secondary malignancies in breast radiotherapy.^6^, ^7^ The integration of auto skin flash tools in RAD further ensured robust superficial target coverage, addressing setup uncertainties without manual bolus adjustments a practical advantage for complex breast geometries and at the same time reduced planning time.

In prostate cases, RAD improved rectal and bladder sparing compared to RA, along with enhanced sparing of femoral heads and bone marrow. Such dosimetric gains align with modern goals of minimizing genitourinary (GU) and gastrointestinal (GI) toxicities,^8^, ^9^ which directly impact long‐term patient quality of life. The ability to strategically embed static fields within arcs likely contributed to these outcomes by refining dose modulation near critical structures.

For lung SBRT, RAD's superior conformity indices and reduced spinal cord, heart, and lung doses highlight its suitability for high‐precision treatments requiring steep dose gradients. The dynamic collimator rotation and embedded static beams in RAD enabled sharper dose fall‐off, critical for preserving central structures while delivering ablative doses to tumors.^10^, ^11^ These technical enhancements may translate to safer dose escalation in challenging SBRT cases.

In head and neck cancer, RAD improved sparing of multiple head‐and‐neck OARs—key predictors of functional outcomes such as salivary function, swallowing, and hearing preservation.^12^, ^13^, ^14^ The ability to pause gantry rotation for optimal beam modulation likely facilitated these improvements, demonstrating RAD's adaptability in anatomically complex regions.

The dosimetric advantages of RAD stem from its advanced delivery features: dynamic collimator adjustments, embedded static fields, and a faster Photon Optimizer algorithm. These innovations allow finer control over dose distributions, particularly for concave targets or tumors near serial OARs. However, these benefits came with increased monitor units (MUs) (e.g., 912 vs. 731 MUs for breast cases). The higher MU count is primarily attributed to the integration of static IMRT fields within arcs, which inherently require additional beam modulation. While a portion of these MUs are blocked by the multileaf collimator (MLC) during delivery—similar to sliding window IMRT techniques—and do not directly contribute to patient dose, the higher MU count observed in RAD plans could theoretically contribute to increased secondary leakage and scatter radiation. However, we did not quantify integral dose or out‐of‐field dose in this study, and therefore this possibility remains speculative. Future work should explicitly assess these parameters to clarify the clinical relevance.

Another practical consideration is planning time. In this study, planning times for RAD were moderately shorter than for RA; however, it is important to note that our clinical team had extensive prior experience with RA and was relatively new to the RAD planning platform. With increasing familiarity and optimization of planning workflows, it is anticipated that RAD planning times could be further reduced.

Despite promising dosimetric outcomes, the clinical relevance of these findings—specifically, whether they translate to reduced toxicities—requires validation through long‐term follow‐up and prospective trials. Additionally, the planning time and complexity associated with RAD warrant further evaluation to optimize workflow integration fully.

This study has several limitations. First, its retrospective nature means that the conventional RA plans were created in clinical settings, while the RAD plans were created specifically for this comparison. This could potentially introduce bias, although we attempted to minimize this by using identical clinical objectives and constraints.

Second, while we demonstrated dosimetric improvements with RAD, these do not necessarily translate directly to clinical benefit. Long‐term follow‐up studies will be necessary to confirm whether the dosimetric advantages result in reduced toxicity rates or improved quality of life.

Third, we did not perform a cost‐effectiveness analysis of implementing RAD, which would require consideration of software/hardware upgrade costs, potential workflow efficiencies, and the monetary value of potential toxicity reductions.

Fourth limitation of this study is that plan verification was not performed. As our center was among the limited sites worldwide participating in the RAD evaluation phase organized by Varian Medical Systems, we only had access to the planning license, while delivery and verification would have required a separate machine license that was not available. Future investigations should incorporate patient‐specific quality assurance and delivery verification to comprehensively evaluate the clinical feasibility of RAD.

Finally, our study focused on specific anatomical sites and may not be generalizable to all clinical scenarios. Additional research is needed to evaluate RAD's performance in other treatment sites and special techniques such as stereotactic radiosurgery or total body irradiation.

## 5 CONCLUSION

In conclusion, this study was designed to compare conventional RapidArc (RA) and RapidArc Dynamic (RAD) across four anatomical sites (breast, prostate, lung SBRT, and head and neck) using identical optimization objectives and dose calculation settings. We found that RAD consistently maintained equivalent target coverage while improving dose conformity and providing superior sparing of critical organs at risk—such as the heart and contralateral lung in breast cancer, rectum and bladder in prostate cancer, spinal cord and heart in lung SBRT, and parotid glands and mucosal structures in head and neck cases. Although RAD plans required higher monitor units, planning efficiency was modestly improved. Taken together, these results demonstrate that RAD represents a meaningful advancement in VMAT delivery, particularly for anatomically complex and high‐precision treatments. Future work should validate these dosimetric advantages in prospective clinical studies and explore the integration of RAD into adaptive radiotherapy workflows.

## AUTHOR CONTRIBUTIONS

Aram Rostami: Co‐authored sections, directed all content, proofing all content, contributed in planning part and analysis, and contributed to writing the manuscript. Carole Naim: Contributed in planning part and writing the manuscript. Renilmon Pottanplackal Sukumaran: Co‐authored sections, contributed in planning part and writing the manuscript. Muhammad Usman: Contributed planning part and proofing all content. Abbass Yousef Mkanna: Contributed planning part and proofing all content. Alla Fuad Al Sabahi: Contributed planning part and proofing all content. Ahamed Basith: Contributed planning part and proofing all content. Shelton Chuck: Contributed planning part and proofing all content. Mojtaba Barzegar: Statistical Analyzes and created figures. Bevan Orville Peltier: Contributed Planning part. Bassim Aklan: Creating all Figures and proofing all content Samaneh Baradaran: Statistical Analyzes and proofing all content. Satheesh Prasad Paloor: Co‐authored sections, proofing all content. Rabih Wafiq Hammoud: Co‐authored sections, directed all content, and co‐developed proofing all content.

## CONFLICT OF INTEREST STATEMENT

The authors declare that this study was conducted independently without any involvement from the vendor. The vendor had no access to patient data, treatment plans, analysis workflows, or the manuscript at any stage. No financial or material support was provided by the vendor for this project. All research activities, data interpretation, and manuscript preparation were carried out solely by the authors.
